# Autonomous Robotic Surgery for Immediately Loaded Implant-Supported Maxillary Full-Arch Prosthesis: A Case Report

**DOI:** 10.3390/jcm11216594

**Published:** 2022-11-07

**Authors:** Shuo Yang, Jiahao Chen, An Li, Ping Li, Shulan Xu

**Affiliations:** Center of Oral Implantology, Stomatological Hospital, Southern Medical University, Guangzhou 510280, China

**Keywords:** guided surgery, implant dentistry, robotic surgery, digital imaging, oral rehabilitation

## Abstract

Robotic systems have emerged in dental implant surgery due to their accuracy. Autonomous robotic surgery may offer unprecedented advantages over conventional alternatives. This clinical protocol was used to show the feasibility of autonomous robotic surgery for immediately loaded implant-supported full-arch prostheses in the maxilla. This case report demonstrated the surgical protocol and outcomes in detail, highlighting the pros and cons of the autonomous robotic system. Within the limitations of this study, autonomous robotic surgery could be a feasible alternative to computer-assisted guided implant surgery.

## 1. Introduction

Successful outcomes of dental implant restorations are mainly determined by precise prosthetically driven implant placement, especially in full-arch implant rehabilitation [1]. Immediately loaded full-arch rehabilitation has been widely accepted by clinicians and patients owing to its high aesthetic and functional expectations [2]. Nevertheless, adequate primary stability is a critical prerequisite for immediately loaded implants, especially in the edentulous maxilla [3]. Considering the low-density bone (type III/IV bone) in the maxilla, a bicortical anchorage of the implants has been suggested to significantly improve the primary implant’s stability [4,5]. Notably, the bicortical stabilization of implants requires a high surgical precision; otherwise, this surgical technique can increase complications, mainly with hemorrhage, nerve injury, and maxillary sinus membrane perforation [6,7].

A precise implant placement can be achieved either by static computer-assisted implant surgery (s-CAIS) or dynamic computer-assisted implant surgery (d-CAIS). The s-CAIS technology uses a surgical implant guide supported by teeth, bone, or mucosa for the drilling process and insertion of the implant [8,9]. Additionally, the d-CAIS systems perform real-time tracking of the drills and implants using an optimal marker [10,11]. Compared to conventional freehand surgery, computer-guided surgery makes dental implant placement predictable, precise, minimally invasive, and efficient [12,13]. Nonetheless, the currently available computer-guided implant surgery still has several limitations [14]. On one hand, the accuracy of s-CAIS reported by a meta-analysis (20 studies) demonstrated the mean deviation at the entry point (1.2 mm), at the apical point (1.5 mm), and the mean angular deviation (3.5°) [8]. Nevertheless, the maximal deviations reported by the two studies were far beyond the acceptable clinical range (<2.0 mm) [9,10]. On the other hand, compared to the s-CAIS systems, the d-CAIS system was highly accurate, with fewer angular deviations [10,11]. However, the drawbacks of the d-CAIS technology not only increase the surgical time and expenditure but also depend on the clinician’s surgical experience [11]. Therefore, development and innovation are necessary for computer-assisted implant surgeries.

Robotic technology has been reported to be a significant innovation in dentistry [15,16]. In this context, a robot-assisted system is commercially available for dental implant surgery. In 2017, the first robotic surgery system (Yomi) was approved by the Food and Drug Administration in the United States [15,16]. As a semi-active robot-assistance system, the haptic robotic guidance consists of a coordinate measurement machine arm and an operational arm, which provide physical (haptic) feedback and visual guidance for the surgeon during implant osteotomy [17]. However, the implant osteotomy is still performed manually by the surgeon using the operational arm [18]. In 2021, an autonomous robot-assisted surgery system called ‘Remebot’ was approved for dental implant surgery by the National Medical Products Administration in China, which was classified as a semi-active and task-autonomy robotic system. The implant osteotomy and placement are automatically performed with image-guided, robot-assisted technologies. Meanwhile, surgeons can monitor the performance of robots during surgery. An in vitro study reported that ten implants were respectively placed in a pig mandible model using an autonomous robotic surgery system. The results demonstrated the coronal deviation (0.69 ± 0.15 mm), the apical deviation (0.72 ± 0.16 mm), and the angular deviation (1.21 ± 0.54°) of the implant surgery using the Remebot surgical robot system with a sufficient clinically accepted range [19]. However, to the best of our knowledge, no clinical case reports are available on the accuracy of dental implant surgery for full-arch implant restoration using the autonomous robotic surgery system.

This clinical case report aimed to demonstrate the feasibility of immediately loaded full-arch implant restorations through a semi-active and task-autonomy robotic system and to determine whether robot-guided precise implant placement results in an appropriate primary implant stability through bicortical fixation.

## 2. Materials and Methods

### 2.1. Initial Status and Treatment Plan

**Figure 1:**
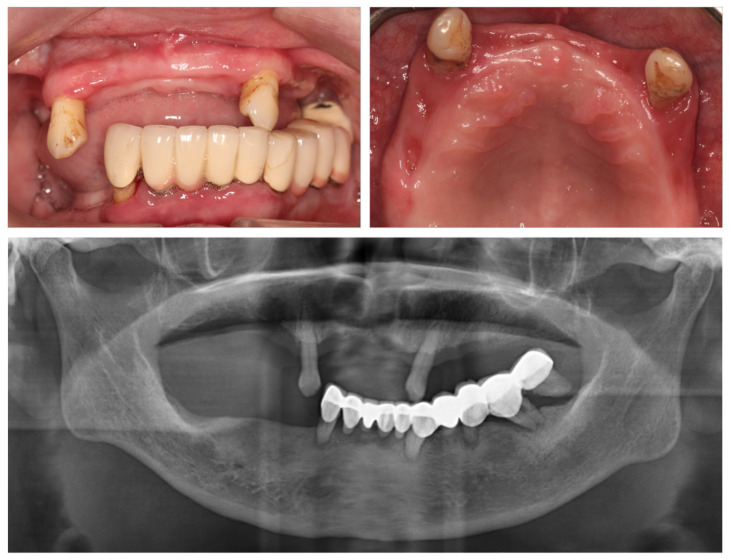
Initial dental status and its panoramic radiograph.

A 58-year-old female was referred to the Center of Oral Implantology, Stomatological Hospital, Southern Medical University (Guangdong Provincial Stomatological Hospital, Guangzhou, China) with a poor masticatory function due to a removable partial denture. The patient had no systemic diseases and was a non-smoker. No apparent abnormalities were detected in extraoral examinations. As shown in Figure 1, the dental status showed some teeth with grade I mobility (teeth #13/23, FDI World Dental Federation notation), partial vertical bone loss, and proper oral hygiene. A poorly designed fixed dental prosthesis was found in the mandible. The initial panoramic radiograph showed a stable crestal bone in the maxilla. Herein, complete-arch fixed implant-supported prostheses were recommended to the patient. However, considering the dental anxiety and financial issues, the patient’s main request was a fixed full-arch prosthesis in the maxilla in a short time. Therefore, based on the patient requirements, the initial treatment plan and primary attention were focused on the upper jaw.

**Figure 2:**
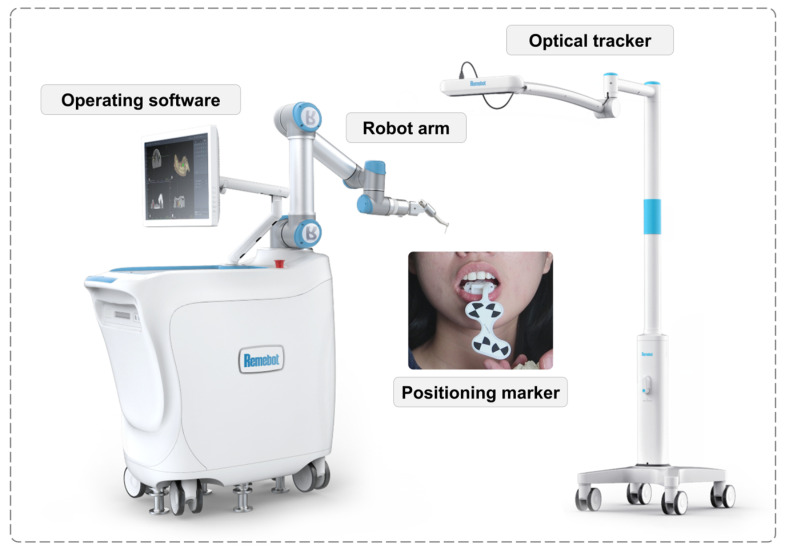
The autonomous robotic surgery system.

After the patient signed an informed consent form, a commercial image-guided robotic oral surgery system (Remebot, Beijing Baihui Weikang Technology Co., Ltd.; Beijing, China) was used to perform immediately loaded full-arch fixed implant rehabilitation. The autonomous robotic surgery system mainly included a robot arm, an optical tracker, a positioning marker, and an operating software system, as shown in Figure 2. The accuracy of the robot arms was provided by the manufacturer, indicating that the average positioning accuracy (trueness) was 0.156 mm (range: 0.071–0.204 mm) and the average repeated positioning accuracy (precision) was 0.033 mm (range: 0.028–0.038 mm). Furthermore, teeth #13/23 were extracted during the implant surgery.

### 2.2. Preoperative Planning

**Figure 3:**
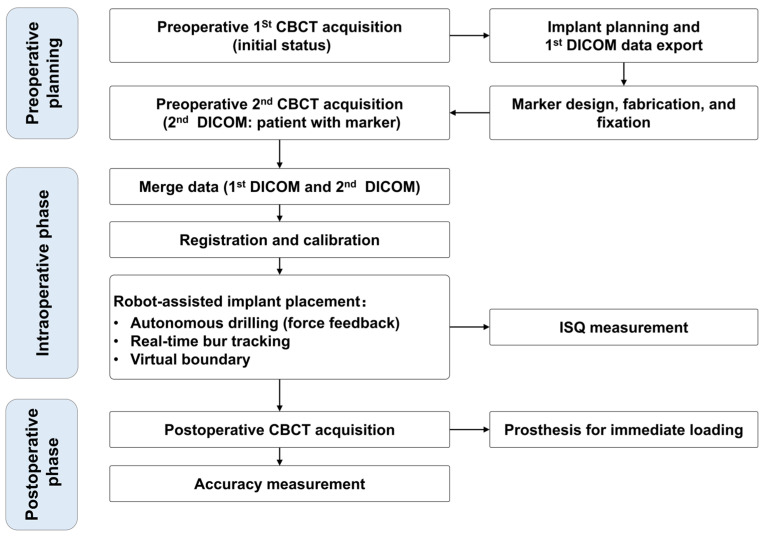
The treatment protocol of robot-assisted implant surgical system for full-arch fixed implant rehabilitation. CBCT, cone-beam computed tomography; DICOM, digital imaging and communications in medicine; ISQ, implant stability quotient.

Figure 3 shows the treatment protocol of the robot-assisted implant surgical system. A video shows the digital workflow for autonomous robotic surgery (Video S1).

**Figure 4:**
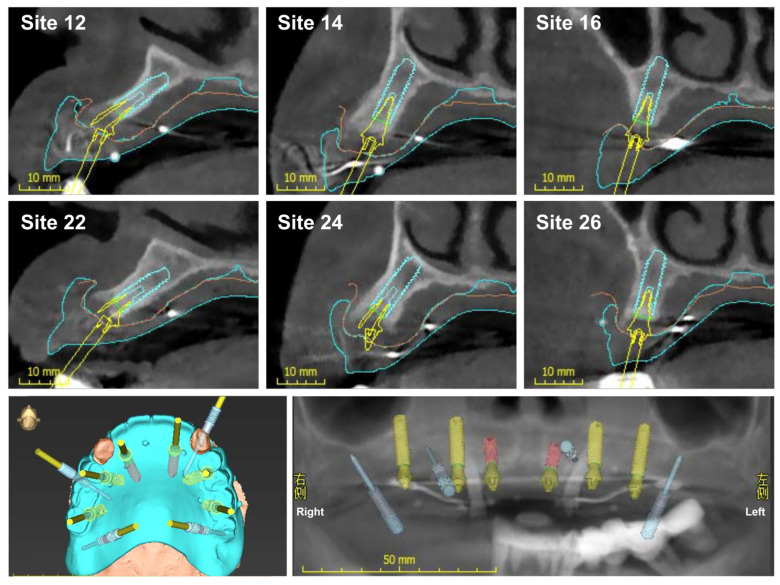
Virtual 3D planning positions for placing six implants and four metallic pins in the maxilla. In the panoramic radiograph, red implants show a diameter of 3.4 mm, yellow implants indicate a diameter of 4.0 mm, and blue ones represent metallic pins.

For the preoperative planning, first, the patient underwent a cone-beam computed tomography (CBCT, NewTom VGI, QR Srl, Verona, Italy) examination with a voxel size of 0.2 mm. The image file was exported to the standard digital imaging and communications in medicine (DICOM) format. Next, the file was input into a virtual implant planning software (coDiagnostiX, Dental Wings GmbH, Chemnitz, Germany). A preoperative full-arch rehabilitation was virtually created. In addition, considering the availability of supporting bone, the implant position was confirmed, and the sites of teeth #16, #14, #24, and #26 were set for bicortical stabilization (Figure 4). Finally, the surgical plan was exported to a DICOM file and termed the first DICOM data.

**Figure 5:**
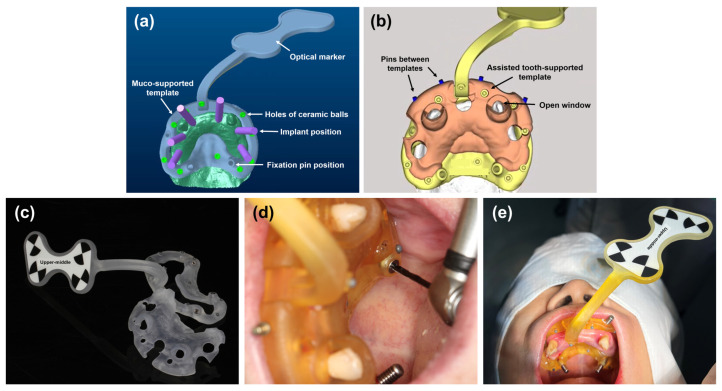
Design, fabrication, and fixation of the customized surgical template with positioning marker. (**a**) Design of the marker template; (**b**) design of the tooth-supported guide template; (**c**) 3D-printed surgical templates; (**d**) the fixation of templates; and (**e**) the patient with the surgical marker template.

A personalized template with a positioning marker was designed using an Exocad software (Exocad GmbH, Darmstadt, Germany), as shown in Figure 5a. An additional tooth-supported guide template was designed to reduce template fixation errors (Figure 5b). Both templates were fixed by metallic pins. A surgical guide material was used to fabricate the templates using a 3D printer (Ultracraft, HeyGears, Guangzhou, China), as illustrated in Figure 5c. The ceramic balls were fixed on the template; then, a positioning marker was calibrated by the manufacturer. The ceramic balls represent the highly radiopaque indicators; the distances between the optical marked point and different ceramic balls were measured and recorded. This facilitated the optical marker’s recognition of three-dimensional structures/spaces through pre-recorded distances. Both surgical templates were placed on the teeth (Figure 5d). Next, the marker template affixation was performed using bone screws under local anesthesia with Primacaine^®^ (4% Articaine, 1:100,000 adrenaline, ACTEON, Mérignac, France). Then, the patient underwent a CBCT examination (Figure 5e), and the second DICOM file with a surgical marker template was exported.

### 2.3. Intraoperative Phase

**Figure 6:**
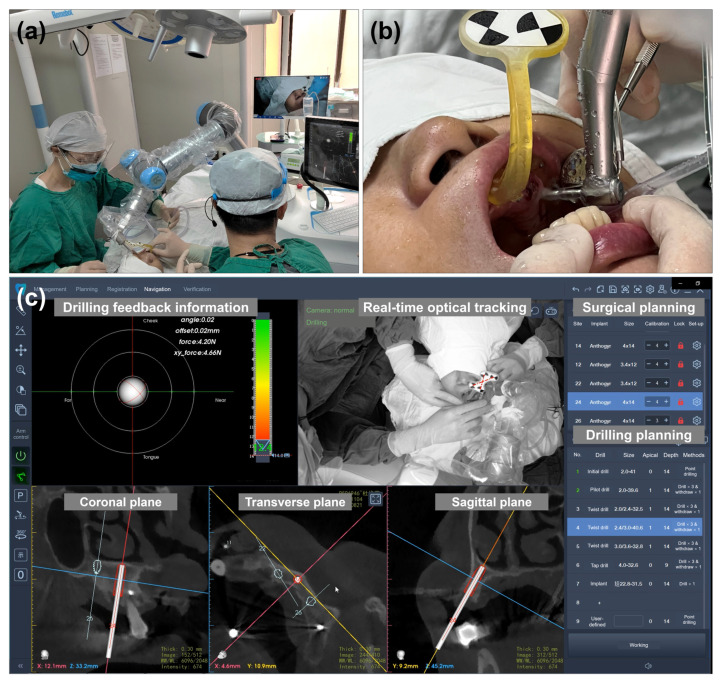
Intraoperative image and autonomous osteotomy procedure. (**a**) The autonomous implant osteotomy was performed by the image-guided robotic surgery system; (**b**) autonomous implant osteotomy at site #24; and (**c**) robot-assisted surgical software system.

Before the surgery, the first DICOM (implant planning) and second DICOM (patient with the positioning marker) were transferred to the robotic surgery system and merged. This protocol could help design prosthetically driven implant placement during the preoperative plan, minimizing the intraoperative time. The 3D areas of interest, such as the alveolar ridge and maxillary sinus, were segmented. The osteotomy plan and target implant position were prepared and visualized. Furthermore, the optical tracker was placed and fixed above the patient’s head. The registration between the positioning marker and the robotic arm was performed. Additionally, the calibration was carried out automatically. The surgeon moved the robotic arm near the oral cavity during the operative phase. Next, the three-dimensional position of the robotic arm was automatically adjusted according to the planned implants. With a flapless procedure, the robotic arm automatically performed the implant osteotomy according to the surgical and osteotomy planning (Figure 6a,b). Based on the real-time surgical system, the surgeon could observe the drilling feedback information (orientation, depth, and force) and real-time drilling position at the coronal, transverse, and sagittal planes (Figure 6c). After the implant site preparation, the dental implants (Axiom Bone Level REG implants, Anthogyr, Sallanches, France) were automatically placed at the sites. When one implant site was finished, the robotic arm was moved to the next site for the implant osteotomy and placement. Then, the implant stability quotient (ISQ) values were measured (Osstell ISQ, Göteborg, Sweden). Finally, the patient underwent a postoperative CBCT scan examination. The abutments were mounted on the implants with a torque of 15 Ncm. The patients received an acrylic resin prosthesis for full-arch rehabilitation connected to implant sites #12, #14, #22, and #24.

### 2.4. Postoperative Accuracy Analysis

**Figure 7:**
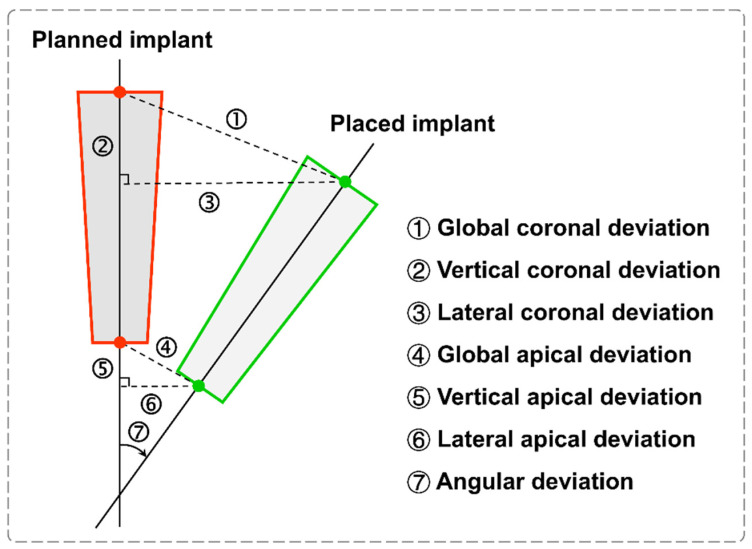
Schematic image of accuracy parameters between the planned and placed implants.

A standardized 3D voxel-based registration for superimposed images of the virtual implant plan and the postoperative CBCT image was used to assess the implant accuracy. Both DICOM files were imported to the robotic surgery verification system, and this registration process was further performed. In addition, the errors between the planned and placed implant positions were measured, as previously described in detail [20,21]. Based on the central axis of the planned and placed implants, the accuracy data showed the distance deviation in mm, including the global coronal deviation, vertical coronal deviation, lateral coronal deviation, global apical deviation, vertical apical deviation, and lateral apical deviation, respectively (Figure 7). Furthermore, the angular deviation was measured in degrees.

## 3. Results

**Figure 8:**
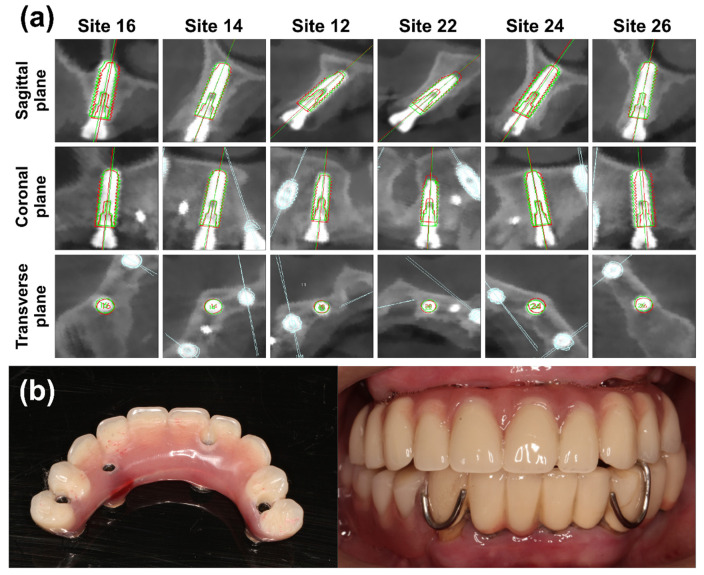
Postoperative evaluation. (**a**) Accuracy analysis for deviations between the planned and placed implants. The red profile indicates the planned implant, and the green profile shows the placed implant; (**b**) the immediately loaded provisional reconstruction.

Six implants were placed in the maxilla. The bone quality ranged from type III to type IV, according to the Lekholm and Zarb classification [22]. The flapless approach was used to treat the arch. No adverse surgical events were reported. As shown in Figure 8, no maxillary sinus membrane perforation or apical bone penetration was observed in the postoperative CBCT images. The profiles of the implants placed at each site matched well with the planned implant. Table 1 lists the accuracy parameters. The means of the global coronal deviation and global apical deviation were 0.59 ± 0.24 mm and 0.61 ± 0.23 mm, respectively. Additionally, the angular deviation was 0.89 ± 0.38 degrees. The mean ISQ value was 73.67 ± 12.71 (range: 55–86). Finally, the maxilla successfully received an immediately loaded full-arch prosthesis (Figure 8).

## 4. Discussion

A precise implant placement is a prerequisite for dental implant restorations to restore esthetics and function and to maintain a healthy performance. Computer-assisted implant surgery has been developed and used to meet clinical requirements [8,23]. However, recent studies demonstrated that the d-CAIS resulted in minor implant placement errors when compared to the s-CAIS [24,25]. Undoubtedly, both static-guided surgery and dynamic-guided surgery have intrinsic drawbacks. The robot-assisted surgical system was introduced to dental implant surgery [26]. Notably, a recent in vivo study via animal experiments reported that implant placement by an autonomous dental implant robotic system was more accurate than that by the s-CAIS [27].

To the best of our knowledge, this clinical case is the first to report the feasibility and accuracy of the immediately loaded implant-supported maxillary full-arch prosthesis through an autonomous robotic surgery system. The clinical results showed relatively lower deviations using the robotic surgery system, achieving bicortical stabilization in the posterior area. Then, the patient received a successful full-arch immediately loaded prosthesis. Accordingly, the overall accuracy of the robotic surgery system met the clinical requirements.

The robotic surgery system’s high accuracy for implant placement can be mainly attributed to the following three factors. First, it is critical to automatically register the surgical and image space in this robotic surgery system before the surgery. Compared to conventional registration, automatic registration and calibration with the optical-system method have higher accuracy, meeting the clinical requirements [28]. Second, regarding freehand surgery, an inaccurate perception and hand tremors could lead to apparent lateral and angular deviation during the implant osteotomy, which is influenced by the surgeon’s experience, implant site, bone quality, etc. [29,30]. On the contrary, the implant site preparation by the autonomous robotic arm can avoid the above individual errors. Third, a positioning marker is required for optical tracking. In this case, a customized tooth-supported guide template with ceramic balls was used (Figure 5b). As expected, rigid tooth-supported fixation proved more reliable than mucosa-supported fixation. Therefore, considering edentulous patients, a mucosa- or bone-supported fixation must be used. However, the accuracy of robotic surgery remains unknown.

Admittedly, the robotic surgery system still has deviations, which can be caused by the following factors. First, the CBCT scan errors were determined by voxel size, exposure time, field of view, and metal artifacts, which influenced the initial data acquisition [31]. Similar to the dynamic dental implant navigation system, the placement and fixation of the marker were affected by the marker type, fabrication, and position using the robotic surgery system [32]. In addition, the robot arm has intrinsic errors, and the average positioning accuracy (trueness) is 0.156 mm, provided by the manufacturer. Finally, considering the automatic implant placement by the robotic surgery system, the non-self-tapping implant could lead to a higher deviation than the self-tapping implant.

The autonomous robotic surgery system is regarded as a novel technology, providing the following main advantages for implant surgery: (1) precise implant osteotomy; (2) where applicable, minimal invasiveness (flapless implant surgery); (3) the efficient avoidance of anatomical risks, such as the alveolar nerve; and (4) real-time feedback on the drilling force to predict the bone quality. However, the main disadvantages of this robotic surgery system should still be considered: (1) an unsatisfactory cost-effectiveness and time-consuming nature; (2) an additional learning curve for the system; (3) an inability to perform implant osteotomy in patients with a limited mouth opening; (4) the optical tracker cannot be moved during surgery; otherwise, the registration should be repeated; and (5) extra radiation: the preoperative CT scans were performed twice in this treatment protocol.

Undoubtedly, a limitation of this case report is that no comparative cohort was enrolled. Additionally, the current treatment protocol can be optimized. For instance, the first CT scan for the template design may be replaced by digital dental impressions to minimize radiation exposure. Meanwhile, implant planning may be performed before surgery. To date, the use of commercially available robotics has not been widely validated in clinical trials, especially in implant dentistry [15,16]. Regarding the application of autonomous robotic surgery systems, there is also a lack of clinical outcomes based on evidence-based medicine. It is suggested that different implant numbers and sites on edentulous arches should be considered using autonomous robotic surgery systems. Therefore, multi-center randomized controlled trials are required.

## 5. Conclusions

This case demonstrated the feasibility of autonomous robotic surgery for immediately loaded implants in the maxilla. Robot-assisted implant surgical systems may open up new horizons for dental implant surgery, enabling accurate and minimally invasive patient-specific procedures. However, further clinical trials are required to provide hard clinical evidence.

## Figures and Tables

**Table 1:** Accuracy parameters and implant stability quotients at each implant site.

Evaluation Parameters	Site 16	Site 14	Site 12	Site 22	Site 24	Site 26	Mean ± SD
Global coronal deviation (mm)	0.61	0.33	0.65	1.03	0.46	0.50	0.59 ± 0.24
Vertical coronal deviation (mm)	0.34	0.27	0.48	−1.01	0.35	0.20	0.11 ± 0.55
Lateral coronal deviation (mm)	0.51	0.19	0.44	0.19	0.30	0.45	0.35 ± 0.14
Global apical deviation (mm)	0.59	0.31	0.56	1.03	0.55	0.61	0.61 ± 0.23
Vertical apical deviation (mm)	0.34	0.27	0.47	−1.02	0.35	0.20	0.10 ± 0.56
Lateral apical deviation (mm)	0.48	0.17	0.30	0.16	0.42	0.57	0.35 ± 0.17
Angular deviation (°)	0.41	1.44	0.87	0.98	0.54	1.09	0.89 ± 0.38
Implant stability quotients (ISQ)	83	81	61	55	76	86	73.67 ± 12.71

## Data Availability

The data presented in this study are available on request from the corresponding author.

## Supplementary Materials

The following supporting information can be downloaded at: https://www.mdpi.com/article/10.3390/jcm11216594/s1; Video S1: the digital workflow for autonomous robotic surgery.
